# Effects of tobacco smoking on cancer and cardiovascular disease in urban black South Africans

**DOI:** 10.1038/sj.bjc.6604303

**Published:** 2008-03-25

**Authors:** L Stein, M I Urban, M Weber, P Ruff, M Hale, B Donde, M Patel, F Sitas

**Affiliations:** 1 MRC/NHLS/Wits Cancer Epidemiology Research Group, National Health Laboratory Service, PO Box 1038, Johannesburg 2000, South Africa; 2 Cancer Epidemiology Research Unit, The Cancer Council NSW, PO Box 572, Kings Cross 1340, Australia; 3 Division of Medical Oncology, Department of Medicine, Johannesburg Hospital and the University of the Witwatersrand, 7 York Road, Parktown, Johannesburg 2193, South Africa; 4 Department of Anatomical Pathology, Chris Hani Baragwanath Laboratory, School of Pathology, University of the Witwatersrand, and National Health Laboratory Service, PO Box 1038, Johannesburg 2000, South Africa; 5 Division of Radiation Oncology, Department of Medicine, Johannesburg Hospital and the University of the Witwatersrand, 7 York Road, Parktown, Johannesburg 2193, South Africa; 6 Clinical Haematology Division, Department of Medicine, Chris Hani Baragwanath Hospital and the University of the Witwatersrand, 7 York Road, Parktown, Johannesburg 2193, South Africa

**Keywords:** tobacco smoking, neoplasms, cardiovascular disease, case–control study, South Africa, domestic fuel

## Abstract

Demographic and lifestyle information from 9690 black patients diagnosed with cancer or cardiovascular disease was collected in an ongoing case–control study in Johannesburg, South Africa. Compared to never smokers, the odds ratio (OR) for lung cancer among current smokers was 16.3 (95% confidence interval (CI), 9.6–27.6) for men and 6.4 (95% CI, 4.0–10.4) for women. The corresponding OR for other smoking-related cancers was 4.6 (95% CI, 3.7–5.7) among men and 1.9 (95% CI, 1.6–2.2) among women, and for cardiovascular disease, 3.4 (95% CI, 2.1–5.4) among men and 1.5 (95% CI, 1.1–2.1) among women. Risks were higher among smokers than former smokers, and all risk estimates increased with increasing levels of smoking duration and intensity. Non-electric domestic fuel was associated with approximately 60% increase in the risk of smoking-related cancer, but not cardiovascular disease. Risks for cancers of cervix, oesophagus, oral cavity/pharynx, stomach, larynx, pancreas and anogenital region, as well as squamous cell carcinoma of skin were all significantly higher among current than never-smokers, with ORs ranging from 1.5 for cervix (95% CI, 1.2–1.8) to 14.7 for larynx (95% CI, 7.2–30). The risks of tobacco-related disease reported here are similar to that currently observed in Western countries, even though cigarette consumption is relatively low in this population.

Certain findings from an ongoing case–control study, which began in 1995, of lifestyle and infectious risk factors for cancer among the black African community of wider Johannesburg, South Africa have been reported (Sitas *et al*, 1999, 2000, 2007; Pacella-Norman *et al*, 2002; Wojcicki *et al*, 2003, 2004; Berrington *et al*, 2006). These include elevated risks associated with tobacco smoking and cancers of the oesophagus, lung, oral cavity and larynx (Pacella-Norman *et al*, 2002) that were broadly consistent with international findings (Vineis *et al*, 2004). The sample size has doubled since the 2002 report, allowing estimates of smoking-related risks of less common cancers (Vineis *et al*, 2004).

The study began just after the implementation of South Africa's first tobacco control legislation and since then both smoking prevalence and overall tobacco consumption has decreased dramatically. In 1995, smoking prevalence among adults was 30.2% and by 2004 it fell to 24.1% (Saloojee, 2006), while in the 10 years to 2001 cigarette consumption had decreased by one-third (Saloojee, 2006). The black African community of South Africa, which accounts for almost two-thirds of the population, has the lowest levels of smoking prevalence within the country (22.7% in 2000, down from 28.1% in 1993) (van Walbeek, 2002a) and consumes the least amount of cigarettes (averaging 7 per day in 1998) (Steyn *et al*, 2002). This partly reflects cultural beliefs and partly poverty on which the excise tax on tobacco has had the greatest effect (van Walbeek, 2002b). The smoking-related cancers of oesophagus, lung, larynx, stomach and cervix remain leading cancer types among black South Africans (Norman *et al*, 2006).

The aim of this analysis was to provide local estimates of the risks associated with smoking for smoking-related cancers and cardiovascular disease, and to explore the effects of duration, intensity and cessation of smoking on these estimates. Because tobacco smoking is associated with several squamous cell cancers (e.g., oral cavity, oesophagus, cervix), we also investigated the squamous cell cancer of the skin (SCC skin), as well as anogenital sites (other than cervix), in relation to smoking.

## MATERIALS AND METHODS

The Johannesburg Cancer Case Control Study is based in the three main public referral hospitals of Johannesburg and Soweto. General methods have been described elsewhere (Sitas *et al*, 1999). In addition, in 1998–2001, patients presenting with cardiovascular diseases were included. The study was approved by the University of the Witwatersrand Human Research Ethics Committee (Medical). At the time of analysis, there were 9690 patients 18 years old or older who had a newly diagnosed cancer or a cardiovascular disease and who were interviewed between March 1995 and June 2004.

In our first series of analyses, the data were arranged into four groups, a control group and three different ‘disease’ case groups. The control group comprised data from 4059 patients who had cancer thought not to be associated with tobacco smoking, and the three case groups comprised patients with (1) lung cancer (423), (2) other cancers known to be associated with tobacco smoking – ‘smoking-related’ (3961), or (3) a cardiovascular disease (716) (Table 1). In a second series of analyses, risks associated with smoking were calculated separately for cancers included in the ‘smoking-related’ group (where *n*>10), as well as anogenital cancers and SCC skin (Table 1).

Altogether, 287 cancer cases were excluded from all analyses because either the cancer was at a secondary site (C77) or the number of cases was small, that is, cancers of the heart (C38), gall bladder (C23), skin (C44 – not SCC), mesothelioma (C45), unspecified genital cancers (C57, C58) and unspecified cancers of the digestive system (C24, C26).

The first series of analyses were stratified by gender and patients were grouped according to particular risk factors, that is, age (18–34, 35–54, 55–98), education (0–5, 6–9, 9+ years), type of domestic cooking fuel (electrical or non-electrical – i.e., wood, charcoal, coal, anthracite, paraffin, or gas), and tobacco smoking status (never, former, current). Since some patients may have given up smoking due to their illness, those who smoked within 5 years of the interview date were classified as current smokers, while those who stopped smoking before this time were classified as former smokers. Never smokers were participants who answered ‘Never’ to the question, ‘Have you ever smoked cigarettes or a pipe regularly?’ Patients were also grouped according to how much tobacco they smoked per day (0, 1–14, 15 g+ assuming weights of 1 g for commercial cigarettes, 1 g for hand-rolled cigarettes, and a conservative 1 g per pipeful), the number of years they smoked (0, 1–20, 21+) and how many ‘pack-years’ they smoked (i.e., number of packs smoked per day multiplied by the number of years smoked; 0, 1–10, 11–20, 21–30, 31+). A ‘pack’ was assumed to contain 20 cigarettes (or 20 hand-rolled cigarettes or 20 pipefuls). As the number of former smokers who smoked more than 30 pack-years was insufficient for analysis, the highest level of pack-years calculated for former smokers was 21 or more.

In the second series of analyses, the data were grouped according to cancer site and distributed by smoking status (never, former, current), but were not stratified by gender due to small numbers of most cancers.

### Statistical analyses

Relative risks for the association of each risk factor and cancer/cardiovascular disease were estimated by using the odds ratios (ORs) derived from unconditional, unmatched multiple logistic regression. A separate term was used for each adjustment factor in the model, and these included: age, education level, and type of domestic cooking fuel. The risk estimates for each individual cancer site in the second series of analyses were also adjusted for gender and alcohol intake (ever/never). A *χ*^2^ test for trend/heterogeneity, adjusting for all other stratification levels, was used to test for significance across exposure levels. Missing variables were added to all regression models as a separate term and are presented in tables as ‘unspecified’.

## RESULTS

The study population was primarily female (65%) with a median age of 52. The median education level was 7 years of schooling (primary school). Many (30%) participants reported current use of non-electric domestic cooking fuel and 41% stated that they had ever smoked, of whom just over one-third had quit 5 or more years prior to interview. Thus, for the purposes of our analyses, 26.6% of the study population were classified as current smokers (13% of women and 52% of men), 14% were former smokers (9% of all women and 24% of all men), and 59.4% as never smokers (78% of women and 23% of men).

Subjects are tabulated by age, education, smoking status and type of domestic cooking fuel in Table 2. Patients with lung cancer were primarily male (79%), over the age of 54 (60%), ‘ever’ smokers (85%), completed a maximum of only 5 years of schooling (68%), and used electricity for cooking (69%). Patients with a smoking-related cancer (other than lung) were primarily male (66%) with ages spread evenly across the 34–54 (46%) and 54–98 age groups (47%), ‘never’ smokers (54%), were schooled for a maximum of 5 years (64%), and 64% used electricity for cooking. Patients with cardiovascular disease were mainly female (78%), with ages spread evenly across the 34–54 (44%) and 54–98 (42%) age groups, with 61% never smokers, 49% with a maximum of 5 years education, and 75% used electricity for cooking.

For both sexes, lung cancer was significantly associated with age and smoking status (Table 2). There was a significant trend in lung cancer risk increasing with age, and with former smokers having a two-fold lower risk than current smokers. Education and type of domestic cooking fuel were associated with lung cancer among men only. Specifically, increased levels of education were associated with a significantly lower risk of lung cancer among men, and lung cancer risk was higher among men who used non-electric cooking fuel.

Smoking-related cancer was significantly associated with age, education, smoking status and type of cooking fuel for both sexes (Table 2). Significant trends in the risk of smoking-related cancer included lower risks among those with higher levels of education, lower risks among former than current smokers, and higher risks among those without electric cooking facilities. Among men, there was a significant trend in the risk of smoking-related cancer increasing with age, but this was not significant among women.

Cardiovascular disease was associated with smoking status for both sexes, with significant lower risk estimates among former smokers than current smokers. Education and domestic cooking fuel were not associated with cardiovascular disease. Age was associated with cardiovascular disease only among men, with participants in the 35–54 years age group at a higher risk than those in the 18–34 years age group.

The use of non-electric domestic cooking fuel was significantly associated with lung cancer (men only) and other smoking-related cancers, but not cardiovascular disease. However, the risk of disease associated with smoking and cooking fuel increased when these two factors were combined. Specifically, ‘ever’ smokers of both sexes increased their risk of a smoking-related cancer or lung cancer by approximately 60% if they also used non-electric cooking fuel; ever smokers among men increased their risk of a cardiovascular disease by 30%. However, among women, this fuel did not increase cardiovascular disease risk above that of ever smokers (Table 2).

The distribution of cases and controls according to number of years smoked, amount of tobacco smoked per day and number of pack-years is shown in Table 3, and each of these variables were significantly associated with all three case groups. There were significant trends across exposure levels for all groups, where risk estimates increased with greater numbers of years smoked, greater amounts of tobacco smoked per day, and with increasing pack-years. Men had higher risks in all the three disease groups than women in terms of all smoking variables analysed. Risks for current smokers across all exposure levels were consistently higher than for former smokers.

**Figure 1:**
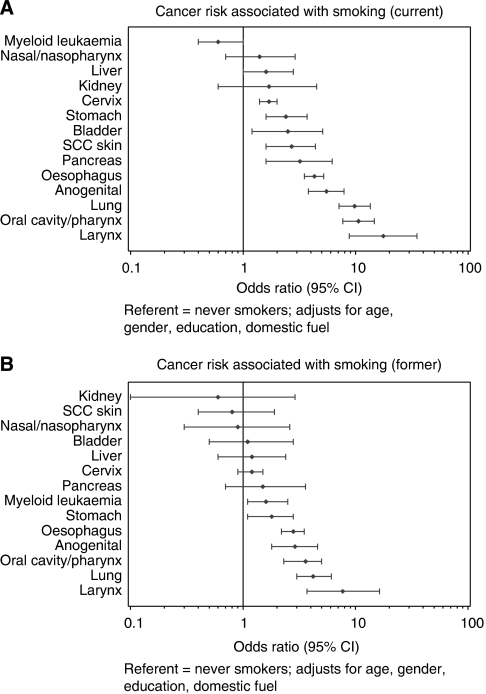
Risks (ORs with 95% CIs) among current (**A**) and former (**B**) smokers for cancer at specific sites in comparison to never smokers. Adjusted for age, gender, education, alcohol consumption, and use of non-electric cooking fuel.

The association between smoking and site-specific cancers is presented in Figure 1. Cancers of cervix, stomach, pancreas, oesophagus, anogenital region (excluding cervix), oral cavity/pharynx, lung, larynx, and SCC skin were all significantly associated with current smoking (Figure 1A). Sites among current smokers that showed nonsignificant elevated risks included the bladder, liver, kidney, and nasal cavity/nasopharynx cancers. In former smokers (Figure 1B), the relative risks were 1.3–3.3 times lower than for current smokers, except for myeloid leukaemia where the risk was significantly higher for former smokers. Relative risk estimates for former smokers remained significantly above those of never smokers for cancers of the stomach, anogenital region, oesophagus, oral cavity/pharynx, lung and larynx, and for myeloid leukaemia.

## DISCUSSION

Using a hospital-based sample of black African patients in Johannesburg, South Africa, this study found elevated risks associated with smoking and cancers of the lung, other smoking-related cancers (i.e., cancers of the cervix, oesophagus, oral cavity and pharynx, stomach, larynx, liver, nasal cavity and nasopharynx, pancreas, bladder, kidney and other urinary sites, and myeloid leukaemia, pooled), and cardiovascular disease, which are similar to international findings (Vineis *et al*, 2004). All smoking-related risk estimates increased with both the number of years smoked and the amounts smoked daily. All risk estimates were significantly lower among former smokers, and were higher overall for men. The use of non-electric domestic cooking fuel was associated with increased risks of lung and other smoking-related cancers, but not of cardiovascular disease. When combined with smoking, the risk of cardiovascular disease was also significantly associated with its use in men, but not in women, and the risk of lung and other smoking-related cancers was further increased for both sexes.

In 2002, the first report on tobacco-related cancer risk from this study was published, comprising just over 4200 participants who were interviewed between 1995 and 1999 (Pacella-Norman *et al*, 2002). With our larger sample size, the risks associated with tobacco smoking reported here are slightly different. Specifically, the relative risk of lung cancer among smokers is now estimated to be higher for men and lower for women when compared to the original report. However, with the increase in the number of lung cancer cases (increased from 110 to 333 in men and 41 to 90 in women), the risk estimates are now more precise.

The 2002 report also investigated an association between non-electric domestic fuel and cancer. With a very small sample size (*n*=6/7), it was found that use of paraffin ‘20 years ago’ for heating was associated with lung cancer in men and oesophageal cancer in women. In the current analysis, we pooled the use of non-electric fuels (including paraffin) and found that a ‘current’ use of non-electric fuel for cooking was associated with a 60% increase in the odds of a smoking-related cancer for both sexes. Non-electric cooking fuel also increased the risk of lung cancer alone by about 60% among men but not in women. Of those reporting a current use of non-electric cooking fuel, 37% used coal, 24% used wood, and 34% used paraffin. The rest were users of gas (4.9%), charcoal (0.2%), or anthracite (0.03%). Residential stoves that use biomass fuels or coal produce extremely high levels of indoor air pollution in the form of particulate matter, nitrogen dioxide, carbon monoxide, and in particular, of carcinogenic polynuclear aromatic compounds such as benzo(a)pyrene, benzene and 1,3-butadiene (IARC, 1985; Cohen *et al*, 2004). Particulate air pollution has been estimated to account for 5% of lung cancer deaths worldwide (Cohen *et al*, 2004), so a significant association of domestic cooking fuel and smoking-related cancers in this study is not surprising.

The larger size of the current study allowed for examination of smoking duration, intensity and cessation. Most men with lung cancer were current smokers who had smoked for over 20 years, and their risk of lung cancer was four-fold higher than smokers of shorter durations. In contrast, most women with lung cancer reported being ‘never smokers’. Nevertheless, current female smokers of more than 20 years had five times the risk of former smokers. There was a 3.5-fold increase in the risk of lung cancer between both men and women who smoked more than 15 g tobacco per day compared to those who smoked less. Furthermore, heavy smoking over a long period yielded a risk of lung cancer that was almost 55 times higher than that of never-smokers in men and 13 times higher in women. However, quitting for at least a 5-year period resulted in a two-fold lower risk of lung cancer overall. A similar pattern of results was found for the risks of smoking-related cancers and cardiovascular disease, where there was a positive dose–response trend for both smoking duration and intensity and an inverse relationship with smoking cessation.

The larger sample size allowed for examination of rarer cancer types. After adjusting for age, gender, education, alcohol intake, and domestic cooking fuel, among current smokers the risk of cancers of the lung, larynx, oesophagus, stomach, pancreas, cervix, anogenital region, oral cavity/pharynx, and SCC skin were higher than among never smokers. The risk of all these cancers was lower among former smokers, however, remained significantly above that of never smokers for cancers of the larynx, lung, oesophagus, oral cavity/pharynx, anogenital region, and stomach. This trend was not apparent for myeloid leukaemia, where the association with smoking was significantly elevated among former smokers but not current smokers; as this is smoking related, it is probably a chance result that may disappear as more cases accrue (The International Agency for Research on Cancer (IARC, 2002).

Almost all of the cancers examined in this analysis were chosen, because they were identified as being partly caused by tobacco smoke in reports from the IARC working group (IARC, 2002; Vineis *et al*, 2004). However, in our study, the relative risk for cancers of the liver, kidney, bladder, and nasal cavity/nasopharynx were elevated but not significantly associated, perhaps because of the (1) small sample sizes; (2) shared exposures between cases and controls other than those used for adjustment (e.g., virus infection, diet, occupation, place of residence); or (3) within this particular population, smoking is not a risk factor for these cancer sites.

Evidence for a relationship of SCC of the skin and cancers of anogenital organs (other than cervix) with smoking remains sparse. A few studies have found smokers to be at a higher risk of cancers of the anus (Daling *et al*, 1992; Northfelt, 1996; Frisch *et al*, 1999; Tseng *et al*, 2003), vulva (Daling *et al*, 1992; Zarcone *et al*, 1997), and penis (Daling *et al*, 1992; Maden *et al*, 1993), but not of the vagina (Daling *et al*, 1992). In our study, current smokers were 4.3 (95% confidence interval (CI), 2.9–6.4) times more likely than never smokers to have one of these cancers (including vaginal cancers). Studies on smoking and SCC skin have had mixed results (Aubry and MacGibbon, 1985; Karagas *et al*, 1992; Grodstein *et al*, 1995; Lear *et al*, 1998; De Hertog *et al*, 2001; Odenbro *et al*, 2005). Current smokers were also at 3.9 (95% CI, 2.1–7.3) times the risk of SCC of the skin of never smokers, which is not dissimilar to estimates in other case–control studies where risks ranged from 2.3 to 3.3 (Aubry and MacGibbon, 1985; De Hertog *et al*, 2001). However, we did not adjust for sun exposure, a primary risk factor for SCC skin, and which is sometimes greater among smokers (De Hertog *et al*, 2001).

Cardiovascular disease showed a two-fold increased risk among smokers, which was approximately two-fold higher than non-smokers; higher than that reported in a national study of tobacco-related deaths (Sitas *et al*, 2004) and in other developing countries such as China (Liu *et al*, 1998). Our risks are similar to those observed in Western countries (Doll *et al*, 2004), although other risk factors for cardiovascular disease, such as diet, were not controlled for; a more comprehensive study incorporating these factors is needed.

The smoking-related risks estimated in this study are likely to be real in the black population of South Africa, as the prevalence of current smokers among controls (18%) is comparable to that reported by the South African Demographic and Health Survey (SADHS) in 1998 (20.4%) (Steyn *et al*, 2002) and in the All Media and Products Survey of the South African Advertising Research Foundation in 2000 (22.7%) (van Walbeek, 2002a). Likewise, the prevalence of former smokers (13%) in controls is similar to that in the SADHS study (6.2% males and 10.3% females) (Steyn *et al*, 2002). However, because we used hospital-based controls, our risk estimates may slightly underestimate the real risks.

The smoking-related risks reported here also indicate the significant burden of disease from smoking in black South Africa, even though tobacco consumption is very low compared to developed countries. Of all 1998 adult deaths (aged >25), 8% were attributed to smoking in an analysis of information on the death notification form (Sitas *et al*, 2004). Smoking prevalence may increase with the growth of the black middle class but is unlikely to reach the levels seen in developed countries in the past, due to peer attitudes and ever more stringent antitobacco legislation (Lopez *et al*, 1994). Case–control studies and analysis of the smoking questions on the national death notification form will allow the burden of tobacco-related disease to be monitored.

## Figures and Tables

**Table 1:** Diseases grouped for analysis

**Site**	**ICD-10**	***n***
*Smoking-unrelated cancers*		
Breast	C50	1442
Prostate	C61	402
Kaposi's sarcoma	C46	384
Colon	C18-C20	333
Non-Hodgkin's lymphoma	C82, C83	239
Endometrium	C54, C55	199
Myeloma	C90	197
Ovaries	C56	196
Hodgkin's disease	C81	156
Leukaemia (not myeloid)	C91-C95	116
Soft tissue sarcoma	C49	105
Bone	C40, C41	71
Melanoma	C43	58
Thyroid	C73	44
Brain	C71	30
Endocrine gland	C75	27
Eye and adnexa	C69	13
Meninges	C70	13
Testes	C62	9
Peritoneum and retroperitoneum	C48	7
Peripheral nerves and ANS	C47	5
CNS	C72	5
Thymus	C37	4
Small intestine	C17	3
Unspecified lymphoid	C96	1
		
*Lung cancer*	C33, C34	423
		
*Squamous cell carcinoma (SCC) of the skin*	C44 (M8070–8076, M8560, M8081, M8051-2)	70
		
*Smoking-related cancers*		
Cervix	C53	1885
Oesophagus	C15	901
Oral cavity and pharynx	C00–C10, C12–C14	369
Myeloid leukaemia	C92	213
Stomach	C16	162
Larynx	C32	139
Liver	C22	88
Nasal cavity and nasopharynx	C11, C30, C31	71
Pancreas	C25	53
Bladder	C67	47
Kidney	C64	25
Unspecified urinary	C68	7
Ureter	C66	1
		
*Anogenital cancers*		
Vulva	C51	107
Anus	C20	31
Penis	C60	22
Vagina	C52	14
		
*Cardiovascular diseases*		
Hypertention	I10–I15	266
Stroke	I64	176
Congestive heart failure	I50	141
Pulmonary heart disease	I26, I27	40
Cerebral infarction	I63	37
Cardiovascular disease	I51	29
Ischaemic heart disease	I24, I25	10
Other vascular diseases	I05–I09, I20, I21, I31, I34,	
	I40, I42, I61, I67, I80	17

**Table 2:** Risk of lung cancer, other smoking-related cancers, and cardiovascular disease distributed by demographic and lifestyle factors^a^

	**Controls^b^**		**Lung Cancer**								**Smoking-related cancer^b^**								**Cardiovascular disease^c^**							
	**Male**	**Female**	**Male**				**Female**				**Male**				**Female**				**Male**				**Female**			
	***n*=1383**	***n*=2676**	***n*=333**		**95% CI**		***n*=90**		**95% CI**		***n*=1330**		**95% CI**		***n*=2631**		**95% CI**		***n*=159**		**95% CI**		***n*=557**		**95% CI**	
**Risk factor**	***n***	***n***	***n***	**OR**	**LCI**	**UCI**	***n***	**OR**	**LCI**	**UCI**	***n***	**OR**	**LCI**	**UCI**	***n***	**OR**	**LCI**	**UCI**	***n***	**OR**	**LCI**	**UCI**	***n***	**OR**	**LCI**	**UCI**
*Age*																										
18–34	253	412	5	1.0	Referent		2	1.0	Referent		90	1.0	Referent		213	1.0	Referent		22	1.0	Referent		77	1.0	Referent	
35–54	478	1212	136	12.5	5.0	31.4	27	2.8	0.6	12.1	530	2.4	1.8	3.3	1283	1.5	1.2	1.8	88	2.0	1.2	3.4	228	0.8	0.6	1.1
55–98	650	1050	192	15.4	6.1	38.7	61	7.1	1.7	30.3	710	2.5	1.9	3.4	1134	1.4	1.1	1.7	49	0.8	0.4	1.5	252	1.0	0.7	1.4
*χ*^2^ (*P* trend)				35.0	(<0.0001)			19.2	(<0.0001)			23.2	(<0.0001)			2.7	(0.10)			2.1	(0.15)			0.5	(0.50)	
Unspecified	2	2	0	—	—	—	0	—	—	—	0	—	—	—	1	—	—	—	0	—	—	—	0	—	—	—
																										
*Education (years)*																										
0–5	761	1317	226	1.0	Referent		61	1.0	Referent		848	1.0	Referent		1683	1.0	Referent		66	1.0	Referent		287	1.0	Referent	
6–8	371	888	87	0.9	0.7	1.2	27	1.1	0.7	1.8	352	0.9	0.8	1.1	728	0.8	0.7	0.9	45	1.2	0.8	1.9	201	1.1	0.9	1.4
9+	243	462	19	0.5	0.3	0.8	2	0.3	0.1	1.2	121	0.7	0.5	0.9	189	0.5	0.4	0.6	27	1.2	0.7	2.1	67	0.7	0.5	1.0
*χ*^2^ (*P* trend)				5.4	(0.02)			1.3	(0.26)			4.6	(0.03)			59.8	(<0.0001)			0.7	(0.41)			1.4	(0.24)	
Unspecified	8	9	1	0.8	0.1	8.0	0	—	—	—	9	1.6	0.6	4.8	31	3.1	1.5	6.6	21	6.1	1.0	36.4	2	1.1	0.2	5.1
																										
*Smoking status* d																										
Never	528	2264	16	1.0	Referent		46	1.0	Referent		176	1.0	Referent		1970	1.0	Referent		25	1.0	Referent		412	1.0	Referent	
Former	338	167	83	7.2	4.1	12.6	12	2.6	1.3	5.1	344	2.8	2.2	3.5	235	1.4	1.1	1.8	23	1.6	0.9	2.8	82	2.6	1.9	3.4
Current	513	227	234	16.3	9.6	27.6	32	6.4	4.0	10.4	808	4.6	3.7	5.7	407	1.9	1.6	2.2	90	3.4	2.1	5.4	63	1.5	1.1	2.1
*χ*^2^ (*P* trend)				142.4	(<0.0001)			58.2	(<0.0001)			189.0	(<0.0001)			50.5	(<0.0001)			29.3	(<0.0001)			15.0	(<0.0001)	
Ever	851	394	317	12.2	7.3	20.6	44	4.6	3.0	7.2	1152	3.9	3.2	4.7	642	1.7	1.4	1.9	113	2.7	1.7	4.3	145	2.0	1.6	2.5
Unspecified	4	18		—	—	—	0	—	—	—	2	2.0	0.3	13.0	19	1.1	0.6	2.2		15.3	2.1	111.7	0	—	—	—
																										
*Cooking fuel* e																										
Electrical	1079	1970	230	1.0	Referent		60	1.0	Referent		937	1.0	Referent		1588	1.0	Referent		104	1.0	Referent		432	1.0	Referent	
Non-electrical	300	703	103	1.6	1.2	2.2	30	1.4	0.8	2.2	390	1.5	1.3	1.9	1030	1.6	1.4	1.8	34	1.2	0.8	1.8	125	0.8	0.6	1.0
*χ*^2^ (*P* heterogeneity)				10.1	(0.002)			1.6	(0.20)			19.4	(<0.0001)			59.6	(<0.0001)			0.5	(0.47)			3.7	(0.05)	
Unspecified	4	3	0	—	—	—	0	—	—	—	3	0.6	0.1	3.4	13	5.1	1.4	18.3	21	5.1	0.7	37.2	0	—	—	—
																										
*Smoking status by cooking fuel*																										
Never smoke/electrical	397	1662	11	1.0	Referent		31	1.0	Referent		113	1.0	Referent		1178	1.0	Referent		17	1.0	Referent		326	1.0	Referent	
Never smoke/non-electrical	131	600	5	1.4	0.5	4.0	15	1.3	0.7	2.4	63	1.6	1.1	2.4	782	1.6	1.4	1.8	8	1.5	0.6	3.6	86	0.7	0.5	0.9
Ever smoke/electrical	681	293	219	11.1	6.0	20.8	29	4.3	2.5	7.3	824	3.9	3.1	5.0	399	1.7	1.4	2.0	87	3.0	1.8	5.2	106	1.8	1.4	2.3
Ever smoke/non-electrical	167	101	98	20.1	10.4	38.9	15	6.8	3.5	13.4	326	6.1	4.6	8.2	241	2.7	2.1	3.5	26	3.6	1.8	7.0	39	1.9	1.3	2.9
*χ*^2^ (*P* trend)				117.8	(<0.0001)			44.6	(<0.0001)			188.0	(<0.0001)			91.6	(<0.0001)			18.1	(<0.0001)			17.9	(<0.0001)	
Unspecified	7	20	0	—	—	—	0	—	—	—	4	2.0	0.5	7.5	31	2.0	1.1	3.6	21	15.8	4.0	62.1	0	—	—	—

a Analyses adjusted for age, education, smoking status, and cooking fuel; odds ratios (OR) with 95% lower (LCI) and upper (UCI) confidence intervals presented.

b See Table 1 for disease groupings.

c Primarily hypertenstion (37%), stroke (25%) and congestive heart failure (18%).

d Current smokers included those who smoked within 5 years of interview.

e Non-electrical cooking fuel included wood, charcoal, coal, anthracite, parafin, and gas.

**Table 3:** Risk of lung cancer, other smoking-related cancers, and cardiovascular disease distributed by smoking variables^a^

	**Controls^b^**		**Lung cancer**								**Smoking-related cancer^b^**								**Cardiovascular disease^c^**							
	**Male**	**Female**	**Male**				**Female**				**Male**				**Female**				***n*=159**				**95% CI**			
	***n*=1383**	***n*=2676**	***n*=333**		**95% CI**		***n*=90**		**95% CI**		***n*=1330**		**95% CI**		***n*=2631**		**95% CI**		***n*=159**		**95% CI**		***n*=557**		**95% CI**	
**Risk factor**	***n***	***n***	***n***	**OR**	**LCI**	**UCI**	***n***	**OR**	**LCI**	**UCI**	***n***	**OR**	**LCI**	**UCI**	***n***	**OR**	**LCI**	**UCI**	***n***	**OR**	**LCI**	**UCI**	***n***	**OR**	**LCI**	**UCI**
*Years smoked*																										
Never smoked	528	2264	16	1.0	Referent		46	1.0	Referent		176	1.0	Referent		1970	1.0	Referent		25	1.0	Referent		412	1.0	Referent	
Former smoker																										
1–20	69	62	15	6.0	3.5	10.4	1	2.6	0.9	7.6	56	2.3	1.6	3.5	58	0.9	0.7	1.4	14	4.3	2.1	8.9	12	1.0	0.6	2.0
21+	53	32	22	10.1	6.0	17.1	29	2.0	0.4	8.5	53	2.8	1.8	4.3	46	1.5	1.0	2.4	7	4.9	1.9	12.5	10	1.5	0.7	3.1
χ^2^ (*P* trend)				49.1	(<0.0001)			2.2	(0.13)			38.2	(<0.0001)			1.1	(0.29)			15.0	(<0.0001)			1.3	(0.26)	
Current smoker																										
1–20	189	59	15	5.1	2.8	9.3	4	1.4	0.2	10.5	98	1.8	1.3	2.5	60	1.2	0.8	1.8	19	1.5	0.7	2.8	14	1.3	0.7	2.4
21+	298	155	210	20.1	14.9	27.2	2	7.4	4.5	12.3	661	6.1	4.9	7.7	319	2.1	1.7	2.5	70	5.5	3.2	9.2	47	1.7	1.2	2.3
χ^2^ (*P* trend)				131.2	(<0.0001)			59.2	(<0.0001)			240.8	(<0.0001)			45.4	(<0.0001)			36.8	(<0.0001)			8.4	(0.004)	
Unspecified	246	104	55	7.1	4.9	10.4	8	3.1	1.4	6.8	286	3.4	2.6	4.3	178	1.7	1.3	2.2	24	0.5	0.2	1.3	62	3.2	2.3	4.4
																										
*Tobacco/day* d																										
Never Smoked	528	2264	16	1.0	Referent		46	1.0	Referent		176	1.0	Referent		1970	1.0	Referent		25	1.0	Referent		412	1.0	Referent	
Former smoker																										
1–14g	243	137	45	5.4	3.0	9.8	11	2.9	1.5	5.8	197	2.3	1.7	2.9	203	1.5	1.2	1.9	15	1.5	0.7	2.9	63	2.4	1.7	3.3
15+g	88	19	36	11.7	6.2	22.2	1	2.0	0.3	15.4	138	4.1	3.0	5.7	25	1.3	0.7	2.3	8	2.1	0.9	4.8	17	4.8	2.5	9.4
χ^2^ (*P* trend)				63.1	(<0.0001)			7.7	(0.006)			100.0	(<0.0001)			10.6	(0.001)			3.9	(0.05)			45.5	(<0.0001)	
Current smoker																										
1–14 g	405	202	113	10.4	6.0	18.0	23	5.3	3.1	9.0	554	4.1	3.3	5.1	353	1.8	1.5	2.2	61	2.9	1.8	4.7	49	1.3	1.0	1.9
15+ g	95	20	115	37.4	21.0	66.5	9	18.5	7.7	44.5	241	6.8	5.1	9.2	50	2.6	1.5	4.5	29	6.1	3.4	11.1	14	3.9	1.9	7.8
χ^2^ (*P* trend)				183.5	(<0.0001)			67.2	(<0.0001)			196.3	(<0.0001)			48.4	(<0.0001)			36.3	(<0.0001)			13.1	(0.001)	
Unspecified	24	34	8	10.2	3.9	26.7	0	—	—	—	24	2.8	1.5	5.2	30	0.9	0.5	1.4	21	1.7	0.4	7.0	2	0.3	0.1	1.4
																										
*Pack years* e																										
Never Smoked	528	2264	16	1.0	Referent		46	1.0	Referent		176	1.0	Referent		1970	1.0	Referent		25	1.0	Referent		412	1.0	Referent	
Former smoker																										
1–10	75	76	18	7.2	3.5	14.8	5	2.5	1.0	6.5	63	2.3	1.6	3.4	76	1.0	0.8	1.5	13	3.5	1.7	7.3	19	1.3	0.8	2.2
11–20	27	9	8	8.4	3.3	21.6	1	3.5	0.4	28.8	19	1.8	1.0	3.4	14	1.6	0.7	3.6	4	4.3	1.3	13.7	2	1.1	0.2	5.2
21+	20	6	11	15.8	6.4	38.7	0	*—*	—	*—*	25	3.4	1.8	6.3	13	2.0	0.8	5.4	4	7.0	2.1	23.4	1	0.9	0.1	7.3
χ^2^ (*P* trend)				46.6	(<0.0001)			2.3	(0.13)			34.7	(<0.0001)			2.5	(0.11)			16.6	(<0.0001)			0.4	(0.52)	
Current smoker																										
1–10	257	140	48	8.1	4.4	14.7	9	3.4	1.6	7.1	257	3.3	2.6	4.2	198	1.5	1.2	1.9	44	3.0	1.7	5.1	35	1.4	0.9	2.1
11–20	117	51	46	11.6	6.3	21.4	9	7.0	3.2	15.3	215	4.9	3.7	6.6	107	2.1	1.5	3.0	15	2.3	1.2	4.7	10	1.1	0.5	2.1
21–30	64	9	42	18.8	9.9	35.7	7	30.0	10.3	87.6	126	5.3	3.7	7.5	44	5.1	2.5	10.5	6	2.1	0.8	5.3	7	4.0	1.5	10.7
31+	41	12	85	54.9	29.3	102.9	5	13.0	4.3	39.3	152	9.5	6.4	14.0	26	2.2	1.1	4.4	24	17.8	8.8	35.9	9	4.0	1.7	9.7
χ^2^ (*P* trend)				190.0	(<0.0001)			64.8	(<0.0001)			188.4	(<0.0001)			44.3	(<0.0001)			43.6	(<0.0001)			14.4	(<0.0001)	
Unspecified	254	109	59	7.0	3.9	12.5	8	3.0	1.3	6.5	297	3.3	2.6	4.2	183	1.7	1.3	2.2	24	0.5	0.2	1.2	62	3.0	2.2	4.2

a Analyses adjusted for age, education, smoking status, and cooking fuel; odds ratios (OR) with 95% lower (LCI) and upper (UCI) confidence intervals presented.

b See Table 1 for disease groupings.

c Primarily hypertenstion (37%), stroke (25%) and congestive heart failure (18%).

d Included cigarettes, hand-rolled cigarettes, and pipes.

e Pack Years calculated by dividing the number of grams of tobacco smoked per day by 20 (1 packet) and then multiplying by the number of years smoked.
