# Effects of tobacco smoking on cancer and cardiovascular disease in urban black South Africans

**DOI:** 10.1038/sj.bjc.6604404

**Published:** 2008-05-05

**Authors:** L Stein, M I Urban, M Weber, P Ruff, M Hale, B Donde, M Patel, F Sitas

**Correction to**: *British Journal of Cancer* (2008) **98**, 1586–1592. doi: 10.1038/sj.bjc.6604303

During the correction process for this article, incorrect versions of the graphs contained in Figure 1 were re-supplied and subsequently published, advance online publication on 25 March 2008 and in this issue.

The corrected Figure 1 is reproduced below.

## Figures and Tables

**Figure 1:**
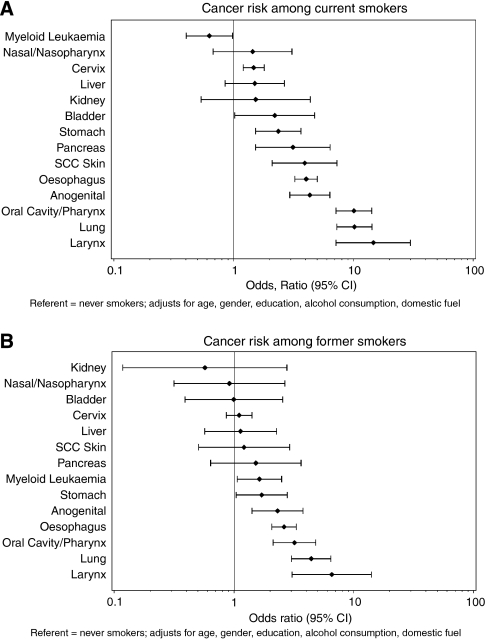
Risks (ORs with 95% CIs) among current (**A**) and former (**B**) smokers for cancer at specific sites in comparison to never smokers. Adjusted for age, gender, education, alcohol consumption, and use of non-electric cooking fuel.

